# Comparative analysis of primary health care indicators security standing in Belgium and Norway: Lessons from readiness to COVID-19

**DOI:** 10.25122/jml-2021-0111

**Published:** 2021

**Authors:** Nazerke Abilkaiyr, Nursymbat Sabyr, Aigul Tazhiyeva, Azimkhan Satybaldin

**Affiliations:** 1. Department of Epidemiology, Biostatistics and Evidence-Based Medicine, Al-Farabi Kazakh National University, Almaty, Republic of Kazakhstan; 2. Faculty of Economics, L.N. Gumilyov Eurasian National University, Nur-Sultan, Republic of Kazakhstan; 3. City Clinic No. 17, Almaty, Republic of Kazakhstan; 4. Institute of Economics, Science Committee of the Ministry of Education of the Republic of Kazakhstan, Almaty, Republic of Kazakhstan

**Keywords:** medical education, therapist, nurse, staff security, comparative analysis

## Abstract

The research aims to suggest the most enabling indicator of COVID-19 resistance in Belgium and Norway by studying the dynamics of staff and bed security indicators of the primary health care sector. The research methodology comprises Organization for Economic Cooperation and Development (OECD) statistical analysis of staff and bed security indicators. The reason for choosing Belgium and Norway for comparative analysis regarding the readiness to face the COVID-19 pandemic in terms of staff and bed security is because Belgium is leading by the highest level and Norway is leading by the lowest level of morbidity and mortality per 1 million population. The study revealed that the greatest enabler of the primary health care system efficiency in terms of resistance to COVID-19 is primary health care staff security. The analysis clearly shows that the number of beds is not paramount for the effectiveness of the healthcare system and primary health care. The COVID-19 pandemic has exposed the deficiencies and weaknesses of primary health care systems of all countries of the world. The research results suggest that Belgium and other countries focus on the education of nurses and therapists. The significance of the research results is that they prove that the main factor of the effectiveness of the primary health care system is its human resources. This information is useful for improving health systems in many countries around the world.

## Introduction

In a condition like the COVID-19 pandemic, the increase in the number of diseases and deaths is a striking indicator of the effectiveness of primary health care. Primary Health Care (PHC) is one of the key elements of the health system, ensuring consistency and continuity of patient interaction, information collection, and disease prevention. World practice shows that an effective PHC system significantly reduces the burden on the rest of the health system, contributes to budgetary savings, and improves the quality of life of the population. That is why the definition of the PHC, its key functions, and organizational characteristics are important in health management [1–2]. Primary health care is based on a commitment to social justice and the recognition of the fundamental right to enjoy the highest attainable standard of health. As reflected in article 25 of the Universal Declaration of Human rights [3]: “Everyone has the right to such a standard of living, including food, clothing, housing, medical care and necessary social services, as is necessary for the health and well-being of himself and his family”.

A review of the existing literature for the subject of interpretation of PHC revealed that many published studies do not define this category, leaving the reader to interpret it differently. This situation is not unique to Russian-language literature. Thus, N. Ramirez *et al.* [4] expatriate scholars, after analyzing more than 2,000 English-language studies on the subject of PHC on the Internet, found that 46% did not include its definitions. This makes it difficult to carry out a theoretical review. Nevertheless, some foreign authors have given “partial” definitions of PHC, implying a “professionally-oriented” approach and an approach oriented to society. Groups of scientists led by F.White *et al.* [5] and R. Martin-Misssener *et al.* [6] in their research follow a “professionally-oriented” approach, usually implying only “clinical contact” and ignoring the role of family members as first-line caregivers and the community has no role in health matters. In contrast, the World Health Organization (WHO) definition applies to the health system as a whole and recognizes the need to involve communities in their health. The WHO concept includes public policy, social and environmental elements in addition to clinical care. The WHO definition reads as follows: Primary health care is a nationwide approach to health and well-being based on the needs and preferences of individuals, families, and communities. The PHC provides for comprehensive life-long care rather than multiple disease management. It ensures that “people receive comprehensive care – from health promotion and disease prevention to treatment, rehabilitation and palliative care – as close as possible to their everyday environment” [7].

As defined by the International Conference on Primary Health Care (Almaty, 1978) [8], PHC is the first level of contact between the population and the national health system. It should be as close as possible to the place of residence and work of people and should constitute the first stage in the continuous process of protecting their health [9]. The definition of B. Starfield is as follows: “PHC is a healthcare approach that goes beyond the traditional healthcare system, which focuses on social policies that ensure health equity” [10]. It should be noted that Canadian law uses the same definition [11]. In some contexts, PHC is understood as the provision of out-patient personal health care or first aid. In other contexts, PHC is understood as a set of priority health interventions for low-income groups (also known as “selective primary health care”). Primary assistance can also be seen as a necessary component of human development, focusing on economic, social, and political aspects [12]. PHC includes all areas that play an important role in health, such as access to health services, the environment, and lifestyles [12].

## Material and Methods

The COVID-19 pandemic has exposed the deficiencies and weaknesses of the healthcare system, in particular, the PHC systems of all countries worldwide. The developed world has been powerless to the easily spreading virus. According to Worldometers [13], on December 12, 2020, the pandemic killed 1.6 million people in the world. However, this is not the case. There were 72.1 million cases of infection registered.

According to world statistics (Table 1), the Top 5 countries in the world are the dwarf and small states of Europe. Also on the first list, the developed countries of Europe and America – Belgium and the United States – stand out. Moreover, on the second list, Belgium leads. For this reason, Belgium was selected for investigation. Norway, the top five countries in Europe with low morbidity rates and Europe’s highest minimal mortality, is selected for background benchmarking from Europe (Table 2).

**Table 1.** The top 10 countries in the world with the highest number of morbidity and mortality per 1 million population [13].

Number of diseases per 1 million population	Number of deaths per 1 million population
1. Andorra – 94.256	1. Belgium – 1.532
2. Luxembourg – 65.457	2. San Marino – 1.502
3. Montenegro – 65.193	3. Peru – 1.101
4. San Marino – 56.885	4. Italy – 1.060
5. French Polynesia – 55.454	5. Spain – 1.018
6. Czech Republic – 53.688	6. Andorra – 1.009
7. Belgium – 51.941	7. Bosnia and Herzegovina – 1.008
8. Bahrain – 51.500	8. Northern Macedonia – 1.006
9. Qatar – 50.156	9. Slovenia – 982
10. United States dollars – 49.866	10. United Kingdom – 941

**Table 2.** Europe's top 10 countries by minimal number of morbidity and mortality per 1 million population [13].

Number of diseases per 1 million population	Number of deaths per 1 million population
1. Isle of Man – 4.341	1. Norway – 71
2. Finland – 5.492	2. Monaco – 76
3. Norway – 7.500	3. Finland – 82
4. Faroe Islands – 10.726	4. Iceland – 82
5. Channel Islands – 11.324	5. Estonia – 112
6. Greece – 11.908	6. Belarus – 133
7. Latvia – 13.346	7. Gibraltar – 148
8. Estonia – 13.349	8. Denmark – 161
9. Ireland – 15.266	9. Latvia – 173
10. Germany – 15.739	10. Slovakia – 210

Across the developed large European countries, Belgium leads by the highest morbidity and mortality rate, and Norway leads by the minimal morbidity and mortality per 1 million population. This is the reason for choosing Belgium and Norway for a comparative analysis regarding readiness to face the COVID-19 pandemic in terms of staff and bed provision. The research methodology comprises OECD statistical analysis of staff and bed provision indicators: hospital employment-to-bed ratio, nurse-to-bed ratio, total hospital beds per 1000 population, practicing physicians per 1.000 population, total hospital beds per 1000 population. The research aims to compare the resource provision of Belgium and Norway in terms of staff and bed and reveal the greatest enabler of primary health care efficiency.

## Results

### Primary health care organization in Belgium

In Belgium, primary health care centers offer quality, accessible and continuous primary care. They employ therapists, nurses, physiotherapists etc. They are part of the so-called “primary health care network” and work together in an interdisciplinary team. They usually receive a fixed per capita payment. Thus, in Belgium, general health care is financed by two different systems: patient consultation fees and a fixed per capita fee for patients who have registered on these service providers [14]. The efficiency of hospital care has improved, but the aim is to strengthen preventive and primary health care. Addressing these challenges requires close coordination between different levels of government. Health expenditure in Belgium has risen steadily over the past 10 years than in most European Union (EU) countries [14]. In 2015, Belgium spent 3.568 euros per capita on health care, compared to the EU average of 2.797 euros. This represents 10.5% of Belgium’s gross domestic product and is above the EU average of 9.9 percent [14]. Public expenditure accounts for 77 percent of total expenditure on health (close to the EU average). The statutory ceiling on public expenditure on health was reduced from a growth rate of 4.5% per annum in 2004-2012 to 1.5% from 2015 [14].

Belgium has a small number of doctors, many nurses, and quotas for medical graduates. As in many other EU countries, Belgium applies a quota system to specialize in general practitioners (GP) and specialist doctors and dentists [14]. Belgium has many different types of hospitals, including general emergency hospitals. The overall hospitalization rate is close to the EU average, as is the average length of hospital stay. The number of hospital beds in Belgium has declined steadily, although it remains higher than the EU average [15]. The provision of health services in Belgium is characterized by the principle of free choice for patients, and primary healthcare doctors are mostly self-employed, working alone and on a fee-paying basis. Since general practitioners have no regular follow-up system, people have free access to specialist doctors and hospital services. Nevertheless, the average number of visits per person does not exceed the EU average [15]. Some features of the healthcare system are contributing to the increased availability of PHC services in Belgium. For example, home visits to patients are common, and patients do not usually wait long to receive a therapist, although waiting times for more specialized services (e.g., mental health professionals, ophthalmologists, dermatologists) may be longer. Nurses also play a key role in providing home-based care services to people with chronic illnesses or disabilities [15].

There is growing concern about the shortage of doctors and other health workers in Belgium. This concern arose between 2004 and 2011 when the number of medical graduates allowed to specialize as physicians or specialists was relatively low. In 2014, 44% of all doctors were over 55 years of age, compared to 24% in 2000, so retirement can be expected in 10 years. In response to these concerns, the Federal Government has steadily increased the quota from 757 seats for 2008-11 to 1.230 seats for 2015-18 since 2011. This is an increase of more than 60% between 2008–2011 and 2015–2018. These postgraduate courses are divided between the Flemish (60%) and French (40%) regions. In addition, the number of interns in general practice increased from a minimum of 300 in 2008-14 to 360 in 2015-18 to better meet the growing demand for primary health care. The number of preventable hospitalizations for chronic diseases, such as asthma, chronic obstructive pulmonary disease (COPD), and diabetes has declined in Belgium over the last decade but is still higher than the EU average [15]. This indicates some shortcomings in the effectiveness of primary health care.

In Belgium, the quality of cancer care, measured by five years of survival for curable cancers, is higher than the EU average. However, cancer mortality can be improved through better prevention and early detection. The coordination of services for cancer patients in hospitals has improved through increased interdisciplinary work. In 2014, total public health and disease prevention expenditure in Belgium accounted for only 2.1% of total health expenditure, which is lower than the EU average (3.0%) [16]. Health promotion campaigns have been developed to reduce smoking, improve nutrition and increase vaccination among target groups. The proportion of people with the lowest income who do not have healthcare for financial, geographical, or waiting reasons increased from about 4% in 2011 to 7% in 2015 [17]. Most of these unmet needs were for financial reasons. Persons entitled to preferential reimbursement of medical expenses are 1) recipients of social benefits, 2) with a taxable gross annual income below a certain threshold, 3) patients with chronic diseases [15–17].

### Resource comparative analysis of primary health care in Belgium and Norway

**Figure 1.**
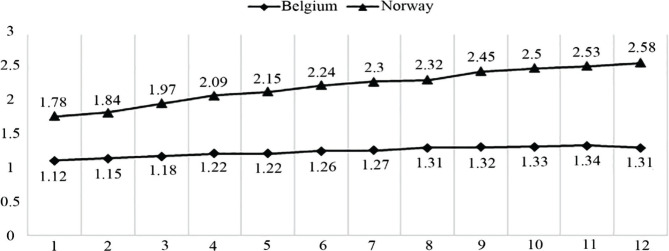
Hospital employment-to-bed ratio (headcounts) [18].

A comparison shows that Belgium lags Norway two times in terms of the ratio of hospital beds (Figure 1) [18]. The increase over the period under review was 32% in Norway and 14% in Belgium. As a result, the gap between countries in this indicator increased by 56% over 12 years. In 2007, it was 2.01%, by 2010, it was 3.14%.

**Figure 2.**
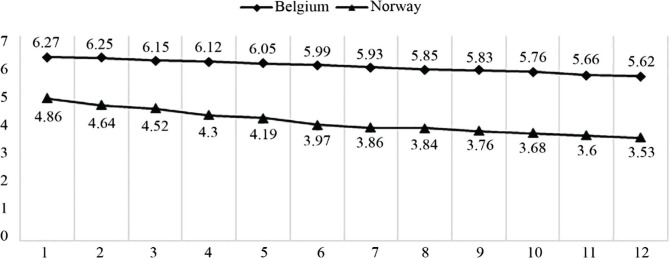
Nurse-to-bed ratio (headcounts) [18].

The ratio of nurses to beds is similar (Figure 2) [18]. The gap between Norway and Belgium by 2018 is 197%, increasing by 38% from 2007. The gap between Norway and Belgium is still very small. The increase during the period under review was 145% for Norway and 117% for Belgium.

**Figure 3.**
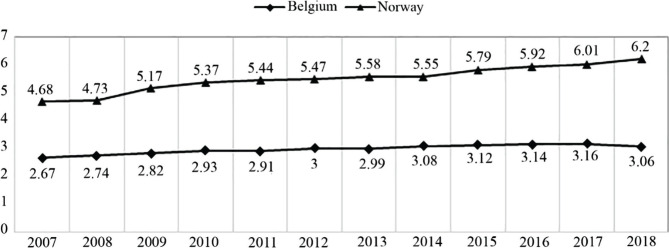
Practicing physicians per 1,000 population (headcounts) [18].

The number of practitioners is an important factor in the effectiveness of the PHC system. The comparison shows that Belgium lags behind Norway (Figure 3) [18]. However, the gap between countries is widening. In 2007, it was 34%, whereas in 2010, the gap was 54%. In 2007, it was 54%. The increase for Belgium was 107.5% and for Norway 123%.

**Figure 4.**
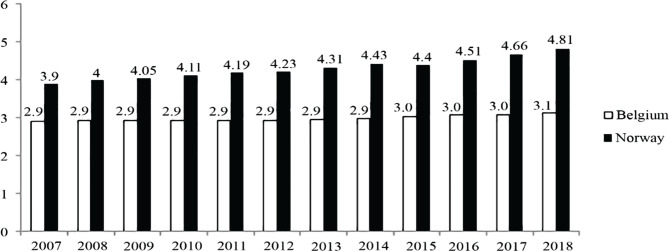
Total hospital beds per 10,000 population [18].

The reverse is true for the population, with Norway lagging behind Belgium by almost 60% (Figure 4) [18]. For both countries, the decline was 11.5% in Belgium and 37.7% in Norway. The higher intensity of the decline in Norway resulted in a doubling of the gap between countries, from 30% in 2007 to 60% in 2018. However, the gap between the two countries increased.

In the 2018 European Health Consumers Rating (EHCI), Belgium and Norway ranked 5^th^ and 3^rd^. The health care system of Belgium is called the “most generous in Europe”. Norway has been rising slowly but steadily in the EHCI ranking for many years. She alone leads on the index “Prophylaxis”. Also, Norway is a leader in indicators “Patient Rights and Awareness” (along with the Netherlands), “Performance” (along with Finland and Switzerland). Perhaps these factors are key in Norway’s leadership in minimal morbidity and mortality from COVID-19 in Europe.

## Discussion

Comparing the situation in Belgium and Norway with the availability of general practitioners, paramedical staff, and hospital beds shows that Norway has a general preference for health personnel, particularly general practitioners, and nurses. The analysis clearly shows that the availability of beds is not a priority for the effectiveness of the healthcare system and PHC. As mentioned earlier, Belgium has a shortage of general practitioners and doctors. In Germany, general practitioners make up almost half of all doctors in the country, and 90% of German citizens have their own home doctor. In the United States, a general practitioner provides 47% of all medical care. In Switzerland, general practitioners represent 73% of all doctors [19–20].

A multidisciplinary approach is essential to the effectiveness of PHC, and it is being actively pursued in many countries. In the United Kingdom, each patient is assigned a specific specialist of a multidisciplinary team [21–22]. That specialist is responsible for creating and maintaining the physical and social activity of the patient, assessing his needs, prioritizing his needs with the patient, providing treatment and rehabilitation within the limits of their competence, and referring to other members of the multidisciplinary team if necessary. In some cases, at the PHC level, there is treatment and control of sufficiently serious diseases, which are usually only diagnosed at the primary care level. For example, Oregon in the United States introduced a cardiovascular disease management model using a multifunctional home team [22].

The team consists of two therapists and their nurses using national guidelines. The criteria for evaluating the team’s effectiveness were the patients’ quality of life, the change in the severity of the disease, the number and reasons of hospitalizations, the satisfaction of the patient. A year later, a study of the model showed that cardiovascular care was effective at the PHC and clinical levels. The study showed no difference, i.e., the mortality of patients treated by multidisciplinary teams of PHC, and patients observed in the clinic was not different [23]. The difference between a general practitioner and a district physician is the greater range of responsibilities and the greater breadth of the medical profile, allowing a timely response to any concern of the patient and collecting more information on his condition. Moreover, the assignment of patients to general practitioners is not based on territorial (i.e., neighborhood) characteristics but on the workload of doctors and the quality of their interpersonal interaction with patients, which improves the quality of health care.

Thus, the study revealed that the main factor in the effectiveness of the primary health care system is its human resources. A comparative analysis showed that Belgium lagged two times behind Norway in terms of the ratio of hospital workers and the number of beds. The gap between countries has increased by 56% in 12 years. In terms of the ratio of nurses to beds, the gap between countries was 197% in 2018. This represents an increase of 38% from 2007. Belgium is also lagging Norway in terms of the number of general practitioners. However, the gap between countries is also widening. By the number of beds per 1000 people, the situation is reversed: negative dynamics are observed across countries, and Norway lags behind Belgium by almost 60%, and this gap is only growing further. Belgium and Norway are ranked 5^th^ and 3^rd^ in the European Health Consumers Rating (EHCI). At the same time, Belgium’s health care system is called “the most generous in Europe”. Norway leads on the index “Prophylaxis”, “Patients’ rights and awareness”, “efficiency”. These factors may be key in Norway’s leadership in the minimal morbidity and mortality from COVID-19 in Europe. A comparative analysis of the situation in Belgium and Norway with the provision of therapists, nurses, and hospital beds indicates the advantage of Norway in the provision of medical personnel in general, particularly therapists and nursing staff. The analysis clearly shows that bed availability is not paramount for the effectiveness of the healthcare system and primary health care. As noted earlier, there is a shortage of therapists and general practitioners in Belgium.

## Conclusion

Based on the above, the authors may conclude that the staffing of PHC is a key factor in the success of the health system, directly influencing the quality of life of the population and the effectiveness of the health system. The PHC combines both a general approach aimed at improving national well-being and specific approaches that personalize medicine to the level of one patient and his or her needs. The PHC plays several roles, not only the curative one but also the coordination of patients in the healthcare system and the collection of all necessary information about them, and the implementation of a whole range of preventive measures, which reduce the number of visits to the education system. 

The historical healthcare system, which includes the community therapist as the key to the PHC due to multiple factors, is now unviable, creating a multitude of unjustified budget and staff hours for specialized healthcare professionals. As a result, the effectiveness of the healthcare system is reduced. A general practitioner (family doctor), who in turn is part of a multidisciplinary team of specialists in disease management as well as in the rest of the time, has become a substitute for a district physician, following the example of developed countries.

## Acknowledgements

### Conflicts of interest

The authors declare that there is no conflict of interest.

### Authorship

NA took part in the concept of the study, design, data collection, data analysis, and interpretation. NS performed data analysis, interpretation, and writing of the article. AT performed design, data analysis, review of the article, and editing. AS was in charge with data collection, interpretation, review of the article, and editing.
